# Nurses have a four-fold risk for overdose of sedatives, hypnotics, and antipsychotics than other healthcare providers in Taiwan

**DOI:** 10.1371/journal.pone.0202004

**Published:** 2018-08-08

**Authors:** Ya-Ting Ke, I-Jung Feng, Chien-Chin Hsu, Jhi-Joung Wang, Shih-Bin Su, Chien-Cheng Huang, Hung-Jung Lin

**Affiliations:** 1 Nursing Department, Chi-Mei Medical Center, Tainan, Taiwan; 2 School of Nursing, Kaohsiung Medical University, Kaohsiung City, Taiwan; 3 Department of Medical Research, Chi-Mei Medical Center, Tainan, Taiwan; 4 Department of Emergency Medicine, Chi-Mei Medical Center, Tainan, Taiwan; 5 Department of Biotechnology, Southern Taiwan University of Science and Technology, Tainan, Taiwan; 6 Department of Leisure, Recreation and Tourism Management, Southern Taiwan University of Science and Technology, Tainan, Taiwan; 7 Department of Occupational Medicine, Chi-Mei Medical Center, Tainan, Taiwan; 8 Department of Environmental and Occupational Health, College of Medicine, National Cheng Kung University, Tainan, Taiwan; 9 Department of Senior Services, Southern Taiwan University of Science and Technology, Tainan, Taiwan; 10 Department of Geriatrics and Gerontology, Chi-Mei Medical Center, Tainan, Taiwan; 11 Department of Emergency Medicine, Taipei Medical University, Taipei, Taiwan; University of Rome Tor Vergata, ITALY

## Abstract

Nurses have high work stress that may contribute to an increased overdose for sedatives, hypnotics, and antipsychotics (OSHA). We conducted this nationwide population-based cross-sectional study to clarify this still unclear issue. We used a nationwide database to identify 110,379 nurses, 22,032 other healthcare providers (HCPs), and an identical number of individuals from the general population matched by age and sex. We compared the period prevalence of OSHA between nurses and the general population, other HCPs and the general population, and nurses and other HCPs, among nurse subgroups from 2006 to 2012. The risk for OSHA in nurses and in the general population was not significantly different after adjusting for anxiety, insomnia, depression, schizophrenia, and affective disorders (adjusted odds ratio [AOR]: 1.145; 95% confidence interval [CI]: 0.974–1.346). However, in the age subgroups < 35 years, nurses had higher risk than the general population of having OSHA (AOR: 1.333; 95% CI: 1.109–1.601). Other HCPs had a significantly lower risk for OSHA than the general population (AOR: 0.237; 95% CI: 0.122–0.460). Nurses had a significantly higher risk for OSHA than other HCPs (AOR: 3.902; 95% CI: 2.159–7.048). Comparison among nurses showed that younger nurses (< 35 years) had a significantly higher risk for OSHA than the older nurses (≥ 50 years) (AOR: 3.569; 95% CI: 1.252–10.330). Registered nurses had significantly higher risk for OSHA than registered professional nurses (AOR: 1.810; 95% CI: 1.405–2.332); and nurses from clinics, local hospitals, and regional hospitals had significantly higher risk than nurses from medical centers. This study delineated that nurses had a nearly four-fold risk for OSHA when compared to other HCPs. Younger nurses, registered nurses, and nurses from clinics, local hospitals, and regional hospitals had higher risks for OSHA than their respective nurse controls; it suggests that more attention should be given to the occupational health of these populations.

## Introduction

Sedatives and hypnotics including benzodiazepines, barbiturates, and nonbarbiturate-nonbenzodiazepine are groups of drugs used for surgical anesthesia, and as treatment for insomnia and anxiety. [1]. Antipsychotics are primarily used to treat delusions, hallucinations, paranoia, or disordered thoughts principally in patients with schizophrenia and bipolar disorder [2]. Most cases of poisoning or overdose of sedatives, hypnotics, and antipsychotics (OSHA) are results of attempted suicide, and may cause mortality or morbidity due to the depression of the respiratory and central nervous systems [1,2]. According to the 2014 Annual Report of the American Association of Poison Control Centers' National Poison Data System, 55,653 single exposures were documented as OSHA. These substances were also at the top four classes (5.9%) of the most frequent substances involved in all human exposures with a mortality of 0.074% [3].

Nursing is a high-stress occupation characterized by excessive workloads, rotating shifts, overtime, and floating to multiple units. These stressors may contribute to substance abuse or overdose [4]. In the USA, it is estimated that about 10% of nurses suffer from drug and alcohol abuse, a statistic similar to that of the general population [4]. The American Nurses Association (ANA) has estimated that 6% to 8% of nurses use either alcohol or drugs to an extent sufficient to impair their professional judgment and patient safety [5]. It has been noted that the use of prescription-type medications including sedatives, hypnotics, and antipsychotics are higher in nurses than in the general population, while marijuana and cocaine use has been noted to be lower in nurses than in the general population [6]. Nurses may have easier access to these medications via their coworkers, such as physicians [6]. In Taiwan, a study reported that benzodiazepine and narcotics were used by 1.8% and 0.7% of nurses, respectively [7]. An Australian coroner between 2003 and 2013 reported that 62.7% of the 404 drug-caused deaths involving healthcare providers (HCPs) were nurses [8]. The most common drugs related to the death were antidepressants/antipsychotics (54.63%) and benzodiazepines (52.54%) [8]. The comparison of OSHA between nurses and individuals in other occupations, however, remains unclear, even after we performed a bibliographic search using the keywords “overdose,” “poisoning,” “addiction,” “sedative,” “hypnotics,” “antipsychotics,” “drug abuse,” “substance abuse,” and “nurse” in the PubMed and Google Scholar databases. Therefore, we conducted this nationwide population-based cross-sectional study to delineate the comparison of OSHA among nurses, other HCPs, and general population.

## Materials and methods

### Data sources

**Fig 1:**
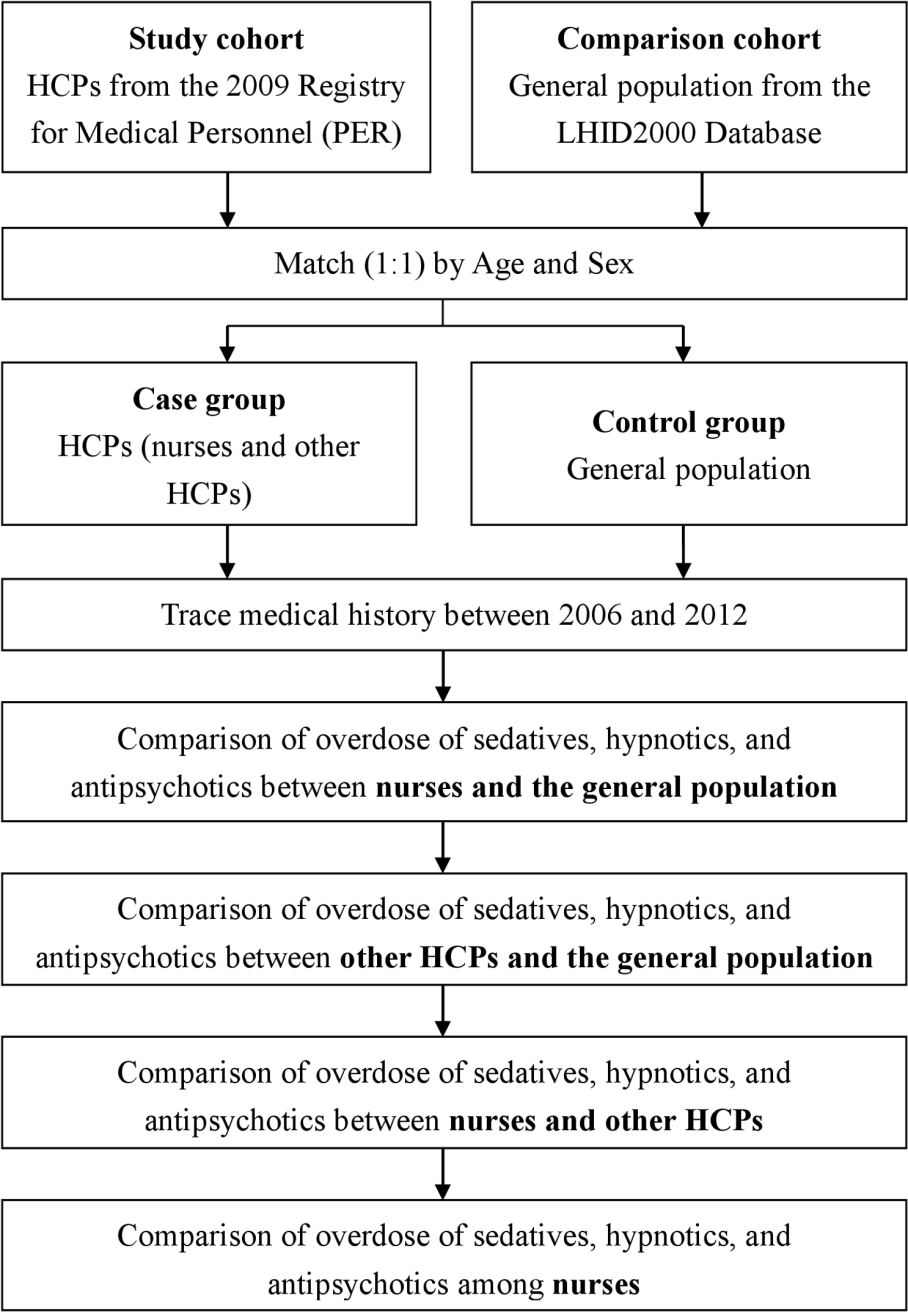
Flowchart of the study. HCP, healthcare provider; LHID, Longitudinal Health Insurance Database.

We used two sub-databases for this study: the 2009 Registry for Medical Personnel (PER) and the Longitudinal Health Insurance Database 2000 (LHID2000), both from the National Health Insurance Research Database (Fig 1). In 2014, the Taiwan National Health Insurance program comprised 99.9% of Taiwan’s population, including foreigners living in Taiwan [9]. The database of this program contains registration files and original claim data for reimbursement [9], and are computerized to serve research purposes for scientists in Taiwan. The data contained in the database are derived from the National Health Insurance Administration (the former Bureau of National Health Insurance, BNHI), the Ministry of Health and Welfare (the former Department of Health, DOH), and are maintained by the National Health Research Institutes, Taiwan [9].

### Identification of the nurses, other HCPs, and general population

For this study, we identified all the nurses and other HCPs who registered in 2009 using the 2009 Registry for Medical Personnel (PER) (Fig 1). We divided the nurses into two categories: registered nurse and registered professional nurse. In Taiwan, both registered nurses and registered professional nurses are licensed nurses. However, the exam for registered professional nurses is limited to those who graduated from a university or five-year college. We identified other HCPs, including medical technologists, physiotherapists, occupational therapists, radiologic technologists, audiologists, counselors, dietitians, social workers, and language therapists to compare alongside the nurses. The fact that these HCPs possess similar socioeconomic backgrounds as nurses may help minimize confounding factors. Physicians were not recruited due to the fact that their socioeconomic backgrounds generally differ from nurses in Taiwan. We matched identical numbers of nurses, other HCPs, and individuals from the general population by age and sex using the LHID2000.

### Definitions of the variables

OSHA was defined by the ICD-9-CM codes 967 or 969 if the participants had either of the above diagnosis one time on admission or three times on ambulatory care. We divided participants’ ages into three subgroups: < 35, 35–49, and ≥ 50, as defined according to a previous study [10]. Comorbidities were defined as anxiety (ICD-9-CM codes: 300, 309.24, excluding 300.4), insomnia (ICD-9-CM codes: 780.52, 307.41, or 307.42), depression (ICD-9-CM codes: 296.2, 296.3, 296.5, 296.82, 300.4, 309.0, 309.1, or 311), schizophrenia (ICD-9-CM code: 295), affective disorders (ICD-9-CM code: 296), hypertension (HTN; ICD-9-CM codes:401–405, A206, A269, 4372), diabetes mellitus (DM; ICD-9-CM codes: 250, A181, A189, A229, A239, 357.2, or 362.0), and were recruited into this study due to the fact that they are risk factors for OSHA [1–8, 10]. Suicide was defined as management codes 94.0 or 94.1, or ICD-9-CM E950-E959 in the index admission or ambulatory care [11].

### Comparison of the risk for OSHA between nurses and general population, other HCPs and general population, nurses and other HCPs, and among nurses subgroups

We compared the risks for OSHA in nurses and the general population, in other HCPs and the general population, and in nurses and other HCPs by tracing their medical history for the diagnosis of OSHA (ICD-9-CM codes 967 or 969) between the period of 2006 to 2012 (Fig 1). Stratified analysis by age, sex, and comorbidities were also performed. We compared nurses and other HCPs because we intended to evaluate the differences in the incidence of OSHA under the similar working conditions of these two populations. Finally, we compared the risk for OSHA among nurse subgroups such as age, sex, classification of nurse, and level of instruction.

### Ethics statement

This study was conducted strictly according to the Declaration of Helsinki and approved by the Institutional Review Board at Chi-Mei Medical Center. Because the databases contain de-identified information, informed consent from the participants was waived. This waiver does not affect the rights and welfare of the participants.

### Statistical analysis

We used independent t-test for continuous variables and the chi-square test for categorical variables in the comparison of demographic characteristics and comorbidities among nurses, other HCPs, and the general population. We used conditional logistic regression by adjusting for anxiety, insomnia, depression, schizophrenia, and affective disorders to compare the risk for OSHA in nurses and the general population and in other HCPs and the general population. Stratified analyses by age subgroups, sex, and comorbidities were also performed. We used unconditional logistic regression analysis by adjusting for age, sex, anxiety, insomnia, depression, schizophrenia, and affective disorders to compare the risk for OSHA between nurses and other HCPs. In the comparison among nurse subgroups, we used unconditional logistic regression analysis by adjusting for all the above variables. We used SAS 9.3.1 for Windows (SAS Institute, Cary, NC, USA) for all analyses. The significance level was set at 0.05 (two-tails).

## Results

### Demographic characteristics and comorbidities of the participants

The mean age of nurses and other HCPs was 31.86years and 33.60 years, respectively (Table 1). Most nurses and other HCPs were females and in the age subgroup < 35 years. Almost all the comorbidities were less frequent in nurses and other HCPs than in the general population, with the exception of HTN, which was found to be not significantly different between nurses and the general population. The suicide rate in the patients with OSHA was 13.73% vs. 17.5% in nurses vs. the general population, and 16.67% vs. 25.71% in other HCPs vs. the general population.

**Table 1:** Demographic characteristics and comorbidities of nurses, other HCPs, and general population.

Variables	Nurses (n = 110379)	General population (n = 110379)	*p*-value	Other HCPs (n = 22032)	General population (n = 22032)	*p-*value
Age, years	31.86 ± 8.32	31.86 ± 8.32	>0.9999	33.60 ± 8.70	33.60 ± 8.70	0.9980
Age, years			>0.9999			>0.9999
<35	79643 (72.15)	79643 (72.15)		13899 (63.09)	13899 (63.09)	
35–49	26842 (24.32)	26842 (24.32)		6957 (31.58)	6957 (31.58)	
≥50	3894 (3.53)	3894 (3.53)		1176 (5.34)	1176 (5.34)	
Sex			>0.9999			>0.9999
Male	1220 (1.11)	1220 (1.11)		7545 (34.25)	7545 (34.25)	
Female	109159 (98.89)	109159 (98.89)		14487 (65.75)	14487 (65.75)	
Comorbidity						
Anxiety	254 (0.23)	1999 (1.81)	<0.0001	30 (0.14)	370 (1.68)	<0.0001
Insomnia	330 (0.3)	1923 (1.74)	<0.0001	29 (0.13)	362 (1.64)	<0.0001
Depression	200 (0.18)	1663 (1.51)	<0.0001	15 (0.07)	326 (1.48)	<0.0001
Schizophrenia	25 (0.02)	135 (0.12)	<0.0001	5 (0.02)	33 (0.15)	<0.0001
Affective disorders	37 (0.03)	85 (0.08)	<0.0001	8 (0.04)	20 (0.09)	0.0376
HTN	316 (0.29)	367 (0.33)	0.0553	76 (0.34)	129 (0.59)	0.0003
DM	158 (0.14)	260 (0.24)	0.0021	32 (0.15)	62 (0.28)	0.0028

HCP, healthcare provider; HTN, hypertension; DM, diabetes mellitus. Data are number (%) or mean ± SD.

### Comparison of overdose of OSHA between nurses and general population and between other HCPs and general population

Overall, there was no significant difference in the risk for OSHA in nurses and the general population (adjusted odds ratio [AOR]: 1.145; 95% confidence interval [CI]: 0.974–1.346) (Table 2). However, in the stratified analysis, it was found that for the age group < 35 years, nurses had significantly higher risk s for OSHA than the general population (AOR: 1.333; 95% CI: 1.109–1.601). Other HCPs had significantly lower risks for OSHA than the general population (AOR: 0.237; 95% CI: 0.122–0.460) (Table 3). Stratified analysis by age subgroup and sex also showed that other HCPs also had lower risks for OSHA than the general population.

**Table 2:** Comparison of overdose of sedative, hypnotics, and antipsychotics between nurses and general population by conditional logistic regression.

	Number (%)	OR (95% CI)	AOR (95% CI)*
Overall analysis			
Nurses (n = 110379)	306 (0.28)	0.850 (0.730–0.990)	1.145 (0.974–1.346)
General population (n = 110379)	360 (0.33)	1	1
Stratified analysis			
Age subgroup			
<35			
Nurses	255 (0.32)	0.996 (0.838–1.185)	1.333 (1.109–1.601)
General population	256 (0.32)	1	1
35–49			
Nurses	48 (0.18)	0.530 (0.374–0.752)	0.751 (0.516–1.092)
General population	91 (0.34)	1	1
≥50			
Nurses	3 (0.08)	0.259 (0.077–0.871)	0.306 (0.085–1.104)
General population	13 (0.33)	1	1
Sex			
Male			
Nurses	2 (0.16)	5.002 (0.121–206.277)	4.218 (0.062–287.834)
General population	0 (0)	1	1
Female			
Nurses	304 (0.28)	0.845 (0.725–0.984)	1.138 (0.968–1.338)
General population	360 (0.33)	1	1
Anxiety			
Nurses	8 (3.15)	0.998 (0.484–2.058)	0.755 (0.323–1.763)
General population	62 (3.10)	1	1
Insomnia			
Nurses	12 (3.64)	1.049 (0.568–1.938)	1.051 (0.547–2.016)
General population	62 (3.22)	1	1
Depression			
Nurses	18 (9.00)	1.416 (0.863–2.322)	1.244 (0.732–2.116)
General population	110 (6.61)	1	1
Schizophrenia			
Nurses	2 (8.00)	1.504 (0.341–6.625)	0.357 (0.018–7.251)
General population	8 (5.93)	1	1
Affective disorders			
Nurses	5 (13.51)	0.901 (0.329–2.470)	0.798 (0.229–2.785)
General population	14 (16.47)	1	1

AOR, adjusted odds ratio; CI, confidence interval.

*Adjusted for anxiety, insomnia, depression, schizophrenia, and affective disorders.

**Table 3:** Comparison of overdose of sedative, hypnotics, and antipsychotics between other HCPs and general population by conditional logistic regression.

	Number (%)	OR (95% CI)	AOR (95% CI)*
Overall analysis			
Other HCPs (n = 22032)	12 (0.05)	0.177 (0.097–0.325)	0.237 (0.122–0.460)
General population (n = 22032)	70 (0.32)	1	
Stratified analysis			
Age subgroup			
<35			
Other HCPs	10 (0.07)	0.231 (0.117–0.454)	0.344 (0.168–0.702)
General population	45 (0.32)	1	
35–49			
Other HCPs	2 (0.03)	0.106 (0.028–0.402)	0.114 (0.019–0.691)
General population	23 (0.33)	1	
≥50			
Other HCPs	0 (0)	0.200 (0.005–8.244)	0.182 (0.001–26.780)
General population	2 (0.17)	1	
Sex			
Male			
Other HCPs	2 (0.03)	0.161 (0.041–0.637)	0.195 (0.045–0.850)
General population	15 (0.20)	1	
Female			
Other HCPs	10 (0.07)	0.189 (0.097–0.368)	0.259 (0.122–0.549)
General population	55 (0.38)	1	
Anxiety			
Other HCPs	1 (3.33)	1.843 (0.301–11.287)	0.725 (0.033–15.955)
General population	9 (2.43)	1	
Insomnia			
Other HCPs	1 (3.45)	1.157 (0.207–6.469)	0.196 (0.009–4.169)
General population	17 (4.70)	1	
Depression			
Other HCPs	1 (6.67)	0.985 (0.183–5.305)	0.220 (0.011–4.324)
General population	26 (7.98)	1	
Schizophrenia			
Other HCPs	1 (20.00)	2.800 (0.270–28.995)	2.012 (0.023–179.959)
General population	2 (6.06)	1	
Affective disorders			
Other HCPs	1 (12.5)	0.913 (0.078–10.668)	0.377 (0.009–16.183)
General population	3 (15.0)	1	

HCP, healthcare provider; AOR, adjusted odds ratio; CI, confidence interval.

*Adjusted for anxiety, insomnia, depression, schizophrenia, and affective disorders.

### Comparison of OSHA between nurses and other HCPs

Nurses had significantly higher risks for OSHA than other HCPs (AOR: 3.902; 95% CI: 2.159–7.048) (Table 4). Stratified analysis showed that nurses had higher risk for OSHA than the general population in the age subgroup < 35 years (AOR: 3.781; 95% CI 1.981–7.211).

**Table 4:** Comparison of overdose of sedative, hypnotics, and antipsychotics between nurses and other HCPs by unconditional logistic regression.

	Number (%)	OR (95% CI)	AOR (95% CI)*
Overall analysis			
Nurses (n = 110379)	306 (0.28)	4.905 (2.786–8.636)	3.902 (2.159–7.048)
Other HCPs (n = 22218)	12 (0.05)	1	1
Stratified analysis			
Age subgroup			
<35			
Nurses	255 (0.32)	4.257 (2.296–7.893)	3.781 (1.981–7.211)
Other HCPs	10 (0.07)	1	1
35–49			
Nurses	48 (0.18)	5.035 (1.412–17.956)	3.209 (0.914–11.264)
Other HCPs	2 (0.03)	1	1
≥50			
Nurses	3 (0.08)	2.116 (0.109–41.041)	0.701 (0.036–13.731)
Other HCPs	0	1	1
Sex			
Male			
Nurses	2 (0.16)	6.191 (1.071–35.787)	4.244 (0.789–22.828)
Other HCPs	2 (0.03)	1	1
Female			
Nurses	304 (0.28)	3.857 (2.084–7.137)	3.778 (2.043–6.986)
Other HCPs	10 (0.07)	1	1
Anxiety			
Nurses	8 (3.15)	0.678 (0.112–4.115)	0.514 (0.091–2.890)
Other HCPs	1 (3.33)	1	1
Insomnia			
Nurses	12 (3.64)	0.746 (0.128–4.348)	0.472 (0.087–2.567)
Other HCPs	1 (3.45)	1	1
Depression			
Nurses	18 (9.00)	0.981 (0.162–5.936)	0.970 (0.169–5.569)
Other HCPs	1 (6.67)	1	1
Schizophrenia			
Nurses	2 (8.00)	0.319 (0.028–3.596)	0.428 (0.030–6.145)
Other HCPs	1 (20.00)	1	1
Affective disorders			
Nurses	5 (13.51)	0.846 (0.106–6.724)	0.758 (0.095–6.058)
Other HCPs	1 (12.50)	1	1

HCP, healthcare provider; AOR, adjusted odds ratio; CI, confidence interval.

*Adjusted for anxiety, insomnia, depression, schizophrenia, and affective disorders.

### Comparison of OSHA among subgroups of nurses

In the comparison among nurse subgroups, younger nurses (< 35 years) had significantly higher risk for OSHA than older nurses (≥ 50 years) (AOR: 3.569; 95% CI: 1.252–10.330) (Table 5). Female nurses had non-significantly higher risk for OSHA than male nurses (AOR: 1.446; 95% CI: 0.416–5.027). Registered nurses had significantly higher risks for OSHA than registered professional nurses (AOR: 1.810; 95% CI: 1.405–2.332). Concerning of the level of instruction, nurses from clinics, local hospitals, and regional hospitals had significantly higher risks for OSHA than nurses from medical centers.

**Table 5:** Comparison of overdose of sedative, hypnotics, and antipsychotics among subgroups of nurses by unconditional logistic regression.

Subgroup	Number (%)	OR (95% CI)	AOR (95% CI)*
Age subgroup			
<35	255 (0.32)	3.578 (1.245–10.281)	3.569 (1.252–10.330)
35–49	48 (0.18)	2.013 (0.680–5.959)	2.018 (0.682–5.974)
≥50	3 (0.08)	1	1
Sex			
Male	2 (0.16)	1	1
Female	304 (0.28)	1.362 (0.392–4.735)	1.446 (0.416–5.027)
Classification of nurse			
Registered professional nurse	224 (0.25)	1	1
Registered nurse	82 (0.41)	1.674 (1.300–2.155)	1.810 (1.405–2.332)
Level of institution			
Medical center	49 (0.16)	1	1
Regional hospital	104 (0.28)	1.732 (1.235–2.430)	1.668 (1.189–2.339)
Local hospital	82 (0.42)	2.637 (1.853–3.753)	2.578 (1.812–3.667)
Clinic	71 (0.32)	2.032 (1.414–2.921)	2.183 (1.519–3.138)

AOR, adjusted odds ratio; CI, confidence interval.

*Adjusted for age, sex, anxiety, insomnia, depression, schizophrenia, and affective disorders.

## Discussion

This nationwide population-based cross-sectional study showed that nurses had no significant difference of risk for OSHA than the overall general population. However, in the age subgroup < 35 years, nurses had significantly higher risks for OSHA than the general population. Other HCPs had significantly lower risks for OSHA than the general population, and stratified analysis by age and sex showed similar results. Nurses had significantly higher risks for OSHA than other HCPs, especially in the age subgroup < 35 years. Younger nurses, female nurses, registered nurses, and nurses from clinics, local hospitals, and regional hospitals had higher risk for OSHA than their respective nurse controls.

Although no significant difference was found in the risk for OSHA in nurses and the overall general population, stratified analysis showed that younger nurses had significantly higher risks for OSHA than the younger general population. In general, nurses were shown to have lower risk for OSHA than the general population due to the healthy worker effect. This effect asserts that workers usually exhibit lower overall mortality or morbidity rates than the general population because sick individuals are ordinarily excluded from employment [12]. This healthy worker effect was also demonstrated in our study when we found that other HCPs had significantly lower risks for OSHA than the general population. However, although considered a healthy worker, nurses in this study had higher risks for OSHA. This suggests that there is indeed an increased risk factor for OSHA in nurses when compared to other occupations.

Higher risk for OSHA in nurses than in other HCPs working in similar environments and under similar conditions, suggests that nurses have more risk factors for overdose than other professionals. A recent study in Australia revealed that nurses predominated (62.87%) the 404 drug-caused deaths involving HCPs [8]. Previous studies about drug abuse or addiction reported that stress induced by conditions such as excessive workloads, rotating shifts, overtime, and floating to multiple units, is the major cause of these conditions [4]. Excessive workload and overtime are caused by the nurse shortage [4,13,14], which has been always a worldwide problem [14]. Insufficient staffing also raises the stress level of nurses, impacts their job satisfaction, and drive many of them to leave the profession [14]. Recent studies in Taiwan found that nurses had higher risk for some kinds of diseases than their coworkers in other healthcare professions. Kuo et al. reported that compared to other HCPs, nurses had significantly higher risks for migraine (AOR: 1.303; 95% CI: 1.206–1.408) [15]. Lin et al. reported that nurses had significantly higher risk for peptic ulcer disease than the general population (OR: 1.477; 95% CI: 1.433–1.521) and other HCPs (OR: 1.052; 95% CI: 1.003–1.102) [16]. Another study showed that nurses also had significantly higher risks for urolithiais than other HCPs (AOR: 1.181; 95% CI: 1.037–1.346) [17]. The authors hypothesized that the characteristics of nursing, including heavy workloads, work stress, shift work, and sleep disturbances are the causes of this condition [15–17].

Our study showed that younger nurses had significantly higher risk for OSHA than older ones, which corroborates a previous study that stated that younger HCPs had higher risk for drug or alcohol abuse than older HCPs [18]. The reasons why younger nurses are more susceptible to OSHA or drug abuse may be due to their lack of experience in confronting stress in work or life and their easy access to drugs via coworkers such as physicians or pharmacists [18]. Registered nurses and nurses from clinics, local hospitals, and regional hospitals had significantly higher risk for OSHA than their respective controls, suggesting that higher educational and knowledge levels may decrease the risk for OSHA. In Taiwan, registered professional nurses have higher educational levels than the registered nurses in general [19]. Nurses in medical centers may always have the opportunity to engage in advanced studies and have easier access for medical knowledge, which may prevent them from OSHA or suffering from drug abuse.

There are some useful strategies for reducing overdose or drug abuse: (1) Educating nursing students or new nurses about the risks and symptoms, of drug abuse and overdose [20]; (2) State boards of nursing addressing this issue at first opportunity with newly licensed nurses [20]; (3) Employers conducting background checks on nurses and addressing the issue of drug abuse in the early stages of hiring [20]; (4) Identifying nurses with these problems and intervening as soon as possible [20]; and (5) Employers a creating an environment where nurses will not be ostracized for drug-related problems and are able to obtain support for recovery [20].

## Limitations

Although this study is the first nationwide study to clarify the comparison of OSHA in nurses and other occupations, it had some limitations. First, this was a cross-sectional study, and may not reflect the real causal relationship between occupations and OSHA. Second, we had no detailed data on the stress levels, lifestyles, and socioeconomic statuses of the participants. This lack prevent us from investigating the associations between these factors and OSHA. Third, although we have done our best to perform a comprehensive search about previous studies, we might have still missed some possibly helpful research on this issue. Fourth, we used ICD-9-CM codes to identify OSHA, which might miss some patients with OSHA. Fifth, the number of male nurses was small, and further studies recruiting more participants are needed to validate the result about this population. Sixth, despite this being a nationwide study, whether or not it can be generalized to other nations needs further clarification.

## Conclusions

This nationwide population-based cross-sectional study delineated that nurses had a nearly four-fold risk for OSHA than other HCPs. Younger nurses (< 35 years) had higher risks for OSHA than their respective younger controls in the general population. Younger nurses, registered nurses, and nurses from clinics, local hospitals, and regional hospitals had higher risks than their respective nurse controls, which suggests that more attention should be given to the occupational health of these populations.
